# The online inverted classroom model (oICM). A blueprint to adapt the inverted classroom to an online learning setting in medical and health education

**DOI:** 10.15694/mep.2020.000113.1

**Published:** 2020-05-28

**Authors:** Daniel Tolks, Bernd FM Romeike, Jan Ehlers, Sebastian Kuhn, Christin Kleinsorgen, Johanna Huber, Martin R Fischer, Christoph Bohne, Inga Hege

**Affiliations:** 1 Institute for Medical Education of the University Hospital; 2 Academic Dean's Office; 3 Didactics and Educational Research in Health Science; 4 Department of Orthopaedics and Traumatology; 5 Centre for E-Learning; 6 Institute for Medical Education of the University Hospital; 7 Brandenburg Medical School Theodor Fontane; 8 Department for Medical Education Sciences

**Keywords:** inverted classroom, flipped classroom, oICM, medical education, health education, digital teaching, synchronous online teaching

## Abstract

This article was migrated. The article was marked as recommended.

The idea of this paper is to offer a blueprint, with that facilitators have a guide to set up a complete digital teaching scenario according to the latest insights of didactical research.

The corona pandemic forced higher education institutions all around the world to radically shift their curricula from a mix of face-to-face and remote teaching methods to a fully remote curriculum. Though challenging, this time provides opportunities to implement new educational methods and improve the quality of digital teaching. The classical concept of the inverted classroom was modified to meet the special needs of online settings. The proposed online Inverted Classroom Model (oICM) includes the following phases: (1) pre-phase, (2) self-learning-phase, (3) Synchronous online face-to-face phase, (4) transfer-phase, (5) evaluation. Recommendations and potential tools are provided for each phase. The oICM is an innovative and easy to use approach to shape digital teaching and learning processes during and after the CoVid19 pandemic. This blueprint is developed by the committee “Digitalization” of the German Association for Medical Education (GMA) for facilitators without any prior experience with the ICM, but also for those who already teach in a traditional ICM.

## 1. Introduction

During the CoVid19 pandemic, educational institutions had to quickly change their curricula to digital teaching. Due to the lack of resources, such as time and experts for digital teaching, the shift in education moved from traditional lectures and seminars to online learning environments. So, we face a state that can be best described by “Emergency Remote Teaching” ( Hodges *et al.*, 2020). This is a challenging time, but it also holds opportunities to improve the quality of digital teaching and learning if applied appropriately. We would like to offer an approach to face the challenge of digital teaching and to implement a new way of online teaching using an existing concept and to modify it to the special needs of this time and the time after the crisis. Thus, we propose a blueprint for the application of the inverted classroom model in an online setting to minimise the disadvantages of online teaching and to achieve real benefits with digital teaching methods.

## 2. The traditional Inverted Classroom Model

According to several meta-analyses, the inverted or flipped classroom method (ICM) showed positive effects regarding engagement, motivation, overall satisfaction and learning outcomes ( Chen, Lui and Martinelli, 2017; Lo, Hew and Chen, 2017; Cheng, Ritzhaupt and Antonenko, 2019; Låg and Sæle, 2019; van Alten *et al.*, 2019; Strelan, Osborn and Palmer, 2020). ICM has successfully been implemented in healthcare education ( McLaughlin *et al.*, 2014; O’Flaherty and Phillips, 2015; Tolks *et al.*, 2016; Chen, Lui and Martinelli, 2017; Hew and Lo, 2018). The idea behind the concept of the inverted classroom proposed by Lage, Platt & Treglia, is to use the face-to-face time for the more challenging part of knowledge application, instead of presenting the factual knowledge in a lecture in which students have to take a passive role ( Lage, Platt and Treglia, 2000). The overall goal is to focus the face-to-face phase on the interactions between students and teacher and to solve problems that may arise during the application of the knowledge. According to Bloom’s taxonomy, the ICM creates a learning environment that enables the learner to reach a higher level of cognition ( Anderson *et al.*, 2013). One of the basic ideas behind the concept is to activate the learner based on the concept of active learning ( Snyder, 2003).

## 3. The online Inverted Classroom Model (oICM)

**Figure 1.**
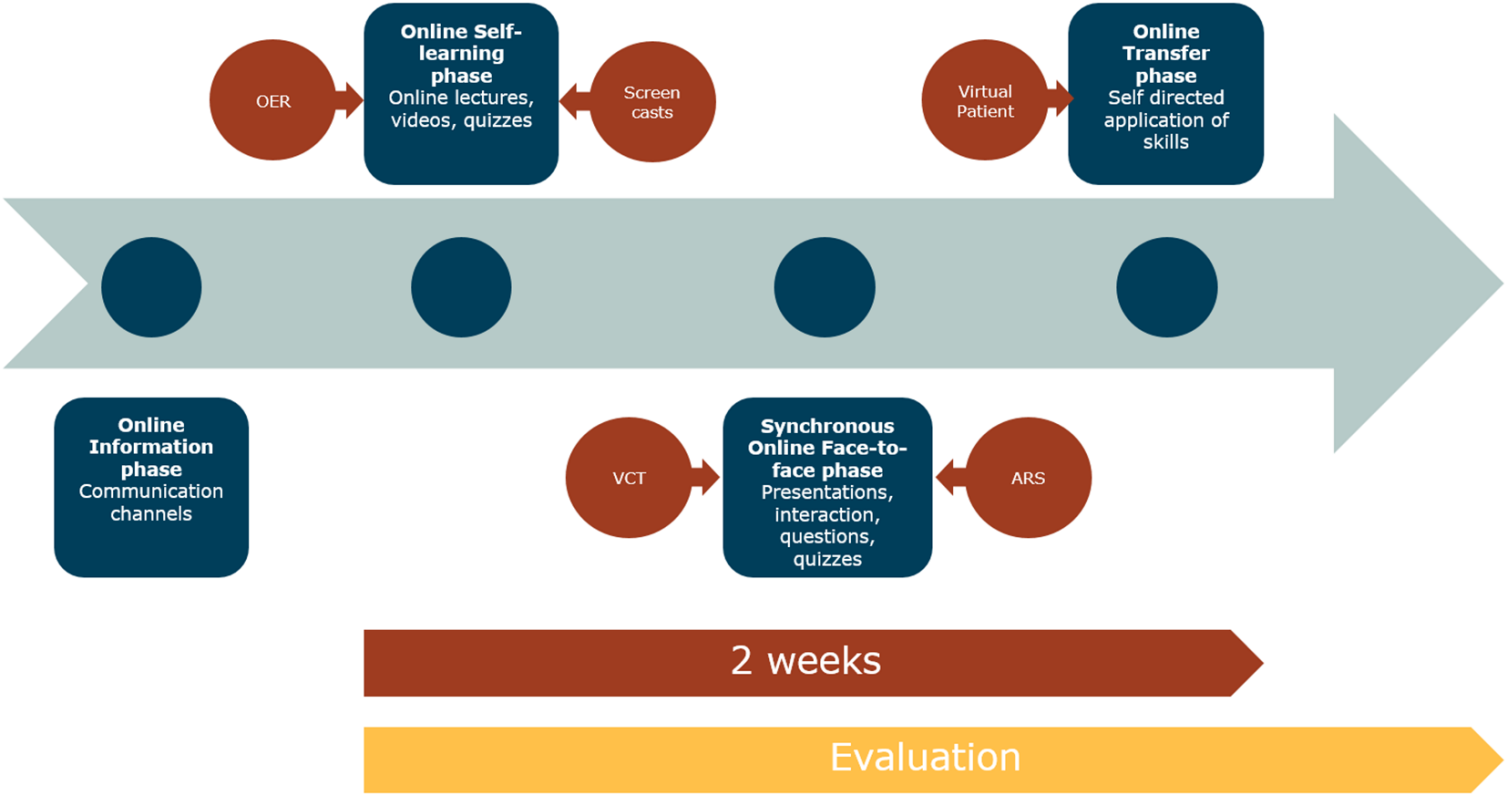
The online inverted classroom model (oICM)

Faced with the challenges of the corona pandemic, universities had to adapt their curricula to online methods quickly. The traditional ICM proposed by Tolks *et al*. has to be adapted to the new approach (see Figure 1) ( Tolks *et al.*, 2016).

This blueprint is designed for teachers without any prior experience with the ICM, but also for those who already teach in a traditional ICM.

Online teaching can be differentiated in synchronous vs. asynchronous delivery modes. In synchronous teaching teacher and learner meet in real-time with online meeting software or video streaming. In asynchronous teaching, learning media is produced and provided via LMS. Students consume contents ad libitum. Advantages and disadvantages of both modes are listed in Table 1.

**Table 1.** Advantages and disadvantages of synchronous and asynchronous delivery modes

	Advantages	Disadvantages
Synchronous	• Instant personal interaction for communication and collaboration • Less isolation • Corporate feeling • Fast reactions to uncover misunderstandings	• Rigid time window • Fast internet connection needed • Sophisticated hardware and software needed • Quiet and acoustically as well as brightness adequate environment needed
Asynchronous	• Media can be downloaded everywhere any time - i.e. flexibility for time and place • Students could spend more time on task - e.g. review material • Permanent availability	• Very limited communication and collaboration • Missing social interaction • Misunderstandings undetected • Huge data • Copyright and privacy violations if media is stored publicly available

As mentioned before, the most important part of the concept is to maintain learner activation. Active learning rather than passivity requires the involvement of students in the learning process, resulting in a more intense learning experience that goes beyond memorization ( Snyder, 2003). Active learning leads to more sustainable knowledge acquisition, promotes problem-solving skills and has a positive effect on the motivation to learn ( Chi and Wylie, 2014). Furthermore, activities and interaction create a more learner-centred environment and are based in constructivist teaching rather than a direct instructional mainly unilateral approach ( Salmon, 2013). Designing well-written learning outcomes for online learning is just as key as for face-to-face environments.

### 3.1 Pre-phase

Before starting to teach, inform your learners about the concept and process of the oICM, technical requirements, and expectations. It is important to offer communication channels for your students. Usually, this will be via your learning management system (LMS) or per e-mail.

### 3.2. Self-learning phase

As for ICM, in this asynchronous phase, it is important to provide learning material that targets the needs of your students. Students are used to learn from videos so we recommend using videos as a source for online learning ( Hurtubise *et al.*, 2013). There are a fast variety of different methods and tools. For some recommendations, you can look at the digital tools list (German) from the committee “Digitalization” of the German Association for Medical Education (GMA): tinyurl.com/ADTLLGMA

There are many ways to create learning content using videos. We would endorse three different approaches.

The easiest way to create great videos is using screencasts. Software allows you to record your presentation together with your speech. You may also include a video of you while talking. These programs are mostly free of charge and easy to use. Be aware of the fact that the presentation does not have to be perfect from a technological and rhetorical point of view. A study by Carpenter *et al*. has shown, that learners have a higher cognitive activity when the presentation is not perfect and that they have a better knowledge retention rate compared to perfect presentations ( Carpenter *et al.*, 2013). Care should be taken to ensure that presentations are not overloaded with content and complex animations, but are structured, high-resolution and reduced to essential elements. This applies especially to online teaching. To support the learning process, a handout could be useful. You can also use existing video tools that help you create innovative learning videos with cartoons (PowTon) or draw images with a digital pencil.

The other possibility is to record the speaker with a camera. This is often used in massive open online courses (MOOCs). These learning videos were analysed in a study by Guo *et al*. regarding the engagement rate of students. To improve learner engagement, the following recommendations can help to improve the quality of the videos ( Guo, Kim and Rubin, 2014):

• Shorter videos are much more engaging
• Videos that intersperse an instructor′s talking head with slides are more engaging than slides alone
• Video produced with a more personal feel could be more engaging than high-fidelity studio screencasts
• Khan-style tablet drawings tutorials are more engaging than Powerpoint slides
• Videos where instructors speak fairly fast and with enthusiasm are more engaging
• Students engage differently with lecture and tutorial videos

A third approach is based on Open Educational Resources (OER). You can always use freely available learning content that is offered under an open-access licence. Many universities offer their high-quality learning material including universities like Oxford, Harvard, Cambridge or the MIT. You can use special databases such as Open Education EU.

In the online self-learning phase, quizzes should be used during and after the learning sessions to improve the learner’s engagement and offer a way to assess the learning process. At the end of the self-learning phase, a final quiz should be implemented to assess the learning status of the group. If any problems or low rates in quiz occur, the teacher can address them in the upcoming online face-to-face phase. Most LMS provide tools for self-assessment.

### 3.3. Synchronous, online face-to-face phase

One way to reduce the limitations of an online scenario such as low retention and engagement rates of students is to use synchronous online meetings. In oICM this phase will take place online using for example video conferencing tools (VCTs), such as AdobeConnect, zoom or goToMeeting. As in ICM the session is moderated by the educator in real time and in an interactive way. Depending on the number of students this can be realized in form of interactive webinars or small group discussions making use of the break out rooms most VCTs offer.

Additionally to VCTs and to engage the learner you can use various digital tools that are easy to use. The most useful tools are audience response systems (ARS) like, Kahoot!, Mentimeter, Pingo. Using those programs, the facilitator can ask quiz questions to the students, create word clouds, rate questions and use gamification approaches such as points, leaderboards and badges ( Sailer and Homner, 2019). The ICM offers a good structure to implement gamification aspects within the learning scenario ( Sailer and Sailer, 2020). Technical questions with the help of ARS continuously activate the participants to collaborate on the content and thus allow a more thorough reflection. While in traditional events potentially more than half of all present participants are mentally distracted, ARS effectively counteracts this. Overall, they have a proven positive effect in learning outcomes ( Nelson *et al.*, 2012; Szpunar, Moulton and Schacter, 2013). In addition, the facilitator receives feedback of a large proportion of those present. Once an interactive quiz is used, learners are more likely to use the chat or audio connection for discussion.

This way, the moderator gets feedback and it lowers the barrier for students to participate actively by talking or using the chat function. Case based learning, clinical reasoning and problem-oriented learning can be easily facilitated by combining an online-meeting with collaborative online documents. For creating complex classifications, students might be asked to build a mindmap.

For students who do not have the opportunity to attend the online face-to-face meeting, the session can be recorded and uploaded to the learning management system (LMS). In this case every participant has to agree to the recording and its use. However, we recommend to include some additional homework to avoid a drop-out of the regular online sessions.

### 3.4. Transfer phase

This phase should deepen the learning outcome with another asynchronous learning session, where the learner can apply their knowledge and transfer it to other content domains or contexts. This can be achieved with small projects students have to work on collaboratively, solving cases or virtual patients or let students develop cases or videos.

## 4. Evaluation

After the oICM, it is also important to evaluate the process and outcome of the curriculum. Especially the online face-to-face phase should be emphasized in the evaluation as it is a new approach within the ICM concept. The evaluation as to be integrated into the online face-to-face meeting to enhance the response rate.

There do already exist some validated or practical tested evaluation forms for traditional teaching units like lectures and seminars ( Perry and Smart, 2007; Schiekirka *et al.*, 2015). However, it is possible to develop a short evaluation form for online ICM based on both, existing instruments of traditional teaching and online teaching. In the Appendix 1, you find some examples for items structured by different aspects of teaching.

## 5. Conclusion

The oICM is based on the traditional ICM, focusing on synchronous digital teaching, the activation of learners and making use of VCTs and audience response systems. The oICM concept supports educators in transferring their previous face-to-face teaching into online teaching in a structured and meaningful way.

An advantage of the oICM concept is that after returning to face-to-face teaching the phases 1, 3 and 4 can be used without changes. Phase 2 can be easily transformed into face-to-face teaching such as small group sessions or seminars including transferring the concept and content for using ARS.

With a few modifications of your existing ICM concept and with this easy-to-use approach, you can significantly improve your digital teaching and do not leave students alone in their learning process. Another positive aspect is that you can still use the ICM even after the pandemic in a blended learning concept. You can continue to use the online learning material provided during the self-learning phase for your teaching concept. The online audience response system can be used in the face-to-face phase to activate the learners. Another advantage is that you will have less issues regarding your teaching load if you use synchronous digital teaching. Before, during and after the CoVid19 pandemic, oICM is an innovative approach to shape digital teaching and learning processes.

We hope that ith this blueprint facilitators will be able to develop own oICM concepts in this critical time and also build concepts that are also feasible for the upcoming time. We would like to encourage all facilitators to invest more time now in the teaching concept and use this blueprint, so that these challenging times have a positive impact to teaching.

## Take Home Messages

• The oICM is an innovative and easy to use approach to shape digital teaching and learning processes during and after the CoVid19 pandemic.
• With a few modifications of an existing ICM concept facilitators can improve their digital teaching and do not leave students alone in their learning process.
• The most important part of the concept is to maintain learner activation with synchronous digital teaching using video conferencing tools and audience response systems.
• The proposed online Inverted Classroom Model (oICM) includes the following phases: (1) pre-phase, (2) self-learning-phase, (3) Synchronous online face-to-face phase, (4) transfer-phase, (5) evaluation.

## Notes On Contributors

**Dr. Daniel Tolks** studied public health and is a researcher at the chair for medical education at the Medical Faculty of LMU Munich and at the Centre for Applied Health Promotion at the Leuphana University Lüneburg. His research interests are technology enhanced learning, gamification and serious games for health. He is chair of the commitee “Digitalization” of the German Association for Medical Education and the German Network Gamification and Serious Games for Health. ORCID iD: https://orcid.org/0000-0001-8597-5189

**Bernd FM Romeike**, MD, MME, is medical educator at the University Medical School Rostock, Germany and clinical neuropathologist. He received his MD in Frankfurt M. in 1994, a habilitation for neuropathology in 2009 at the Homburg Medical School, and a Master of Medical Edication in Heidelberg in 2017. ORCID iD: https://orcid.org/0000-0002-9693-3870

**Jan P. Ehlers**, DVM, MA, FTA, is a veterinarian, instructional designer and medical educator. Her holds the chair for didactics and educational research in healthcare at the medical department and serves as vice president of Witten/Herdecke University, Germany. His research interests are digital transformation of health care, technology enhanced learning and higher education didactics. ORCID iD: https://orcid.org/0000-0001-6306-4173

**Sebastian Kuhn**, MD, MME is a Orthopedics and Trauma surgery and Medical Educator. His research interest on digital transformation an artificial intelligence in healthcare and education. ORCID iD: https://orcid.org/0000-0002-8031-2973

**Dr. Christin Kleinsorgen**, is a veterinarian and research associate in the Centre for E-Learning, Didactics and Educational Research at the University of Veterinary Medicine in Hannover, Germany. ORCID iD: https://orcid.org/0000-0003-1086-1691

**Johanna Huber**, MPH is a research associate and post-doc researcher at the chair for medical education at the Medical Faculty of LMU Munich and works in the field of evaluation studies, questionnaire construction and validation, graduate studies with a focus on the scientific, professional and social skills development, and health research capacity development.

**Martin Fischer**, MD, MME, FAMEE, is an internist, endocrinologist, and medical educator. He holds the chair for medical education and serves as the Assoc. Dean of Clinical Studies at the Medical Faculty of LMU Munich, Germany. His research interests are clinical reasoning skills, faculty and curriculum development, and technology-enhanced learning. ORCID iD: https://orcid.org/0000-0002-5299-5025

**Christoph Bohne** is a research scientist and specialist for educational technology at the Brandenburg Medical School Theodor Fontane.

**Inga Hege**, MD, MCompSc, is an Associate Professor for Medical Education at the Medical School, University of Augsburg, Germany. ORCID iD: https://orcid.org/0000-0003-4335-5162

## Appendices

### Appendix 1. Evaluation form

**Table:**

Aspect of teaching	Items	Scale/ Answer format
Organization and technology	It was clearly communicated from the beginning how the online course format will look like.	5 Point Likert Scale for agreementn.a.
	Technical problems occurred before or during the teaching session.	5 Point Likert Scale for agreementn.a.
	If yes, please briefly describe the technical problems:	Free text question
Learning content	Which online activities (forms of learning/learning materials) were used in the teaching session?	• Online communication (e.g. Forum) • Live lesson (e.g. via Zoom or Adobe Connect) • Self-learning test (e.g. MC questions, AMBOSS question sessions) • Instructional video Podcast (audio record) • Script (e.g. PPTslide sets, summaries) • Online (group) task
	I have participated in live lessons or online communication (e.g. forum).	• Completely • Partially • Not at all • n.a.
	I have used/worked with the provided online learning materials.	• Completely • Partially • Not at all • n.a.
	The learning content of the provided materials was understandable.	5 Point Likert Scale for agreement n.a.
	Which online activities were particularly helpful for you and why?	Free text question
Didactics and support	The learning goals that I was supposed to achieve were clarified at the beginning.	5 Point Likert Scale for agreement n.a.
	The online activities in this teaching session had clear tasks and goals.	5 Point Likert Scale for agreement n.a.
	During online activities the teacher supported me well.	5 Point Likert Scale for agreement n.a.
	The lecturer responded well to questions and suggestions.	5 Point Likert Scale for agreement n.a.
	I was encouraged to critically reflect upon the contents taught.	5 Point Likert Scale for agreement n.a.
4. Learning success	I can give an overview of the contents of the teaching session.	5 Point Likert Scale for agreement n.a.
	Measured by my previous knowledge, I learned a lot during the teaching session.	5 Point Likert Scale for agreement n.a.
Overall rating	I particularly liked the following aspects of the teaching session:	Free text question
	The following three aspects should be improved:	Free text question
	Overall, I rate the attended teaching session with:	5 Point Scale: excellent to insufficient n.a.
