# The online inverted classroom model (oICM). A blueprint to adapt the inverted classroom to an online learning setting in medical and health education

**DOI:** 10.15694/mep.2020.000113.2

**Published:** 2021-09-29

**Authors:** Daniel Tolks, Bernd FM Romeike, Jan Ehlers, Sebastian Kuhn, Christin Kleinsorgen, Johanna Huber, Martin R Fischer, Christoph Bohne, Inga Hege, Luisa Merz, Michael Sailer

**Affiliations:** 1 Institute for Medical Education of the University Hospital; 2 Institute for Medical Education of the University Hospital; 3 Academic Dean's Office; 4 Academic Dean's Office; 5 Didactics and Educational Research in Health Science; 6 Didactics and Educational Research in Health Science; 7 Department of Orthopaedics and Traumatology; 8 Department of Orthopaedics and Traumatology; 9 Centre for E-Learning; 10 Centre for E-Learning; 11 Institute for Medical Education of the University Hospital; 12 Institute for Medical Education of the University Hospital; 13 Brandenburg Medical School Theodor Fontane; 14 Brandenburg Medical School Theodor Fontane; 15 Department for Medical Education Sciences; 16 Department for Medical Education Sciences; 17 Center for International Health CIH; 18 Education and Educational Psychology

**Keywords:** inverted classroom, flipped classroom, oICM, medical education, health education, digital teaching, synchronous online teaching

## Abstract

This article was migrated. The article was marked as recommended.

The idea of this paper is to offer a blueprint, to guide educators setting up a complete digital teaching scenario according to the latest insights of educational research.

The COVID-19 pandemic forced higher education institutions all around the world to radically shift their curricula from a mix of face-to-face and remote teaching methods to a fully remote curriculum. Though challenging, this time provides opportunities to implement new educational methods and to improve the quality of digital teaching. The concept of the inverted classroom was modified to meet the special needs of the new online settings. The proposed online Inverted Classroom Model (oICM) includes the following phases: (1) pre-phase, (2) self-learning-phase, (3) synchronous online phase, (4) transfer-phase, and (5) evaluation. Recommendations and potential tools are provided for each phase. The oICM is an innovative and easy to use approach to shape digital teaching and learning processes during and after the COVID-19 pandemic. This blueprint is developed by the committee “Digitalization” of the German Association for Medical Education (GMA), mainly for educators who are familiar with the Inverted Classroom Model (ICM) or similar blended learning formats.

## 1. Introduction

During the CoVid19 pandemic, educational institutions had to quickly adapt their curricula to digital teaching. Despite the lack of resources, such as time and experts for digital teaching, the shift in education moved from traditional lectures and seminars to online learning environments. Hence, we face a state that can be best described by “Emergency Remote Teaching” ( Hodges *et al.*, 2020). This is a challenging time, but it also holds opportunities to improve the quality of digital teaching and learning if implemented appropriately. We would like to offer an approach to face the challenge of digital teaching and to implement a new way of online teaching using an existing concept and modifying it to the special needs of this time plus the time after the pandemic. Thus, we propose a blueprint for the application of the inverted classroom model (ICM) in an online setting to minimize the disadvantages of online teaching and to achieve real benefits with digital teaching methods.

## 2. The traditional Inverted Classroom Model

According to several meta-analyses, the inverted or flipped classroom method (ICM) showed positive effects regarding engagement, motivation, overall satisfaction, and learning outcomes ( Chen, Lui and Martinelli, 2017; Lo, Hew and Chen, 2017; Cheng, Ritzhaupt and Antonenko, 2019; Låg and Sæle, 2019; van Alten *et al.*, 2019; Strelan, Osborn and Palmer, 2020). ICM has successfully been implemented in healthcare education ( McLaughlin *et al.*, 2014; O’Flaherty and Phillips, 2015; Tolks *et al.*, 2016; Chen, Lui and Martinelli, 2017; Hew and Lo, 2018). The idea behind the concept of the inverted classroom is to use the face-to-face time for the more challenging part of knowledge application, instead of presenting the conceptual knowledge in a lecture in which students have to take a passive role ( Lage, Platt and Treglia, 2000). The overall goal is to focus the face-to-face phase on the interactions between students and teacher and to solve problems that may arise during the knowledge application. According to Bloom’s taxonomy, the ICM creates a learning environment that enables the learner to reach a higher level of cognition ( Anderson *et al.*, 2013). One of the basic ideas behind the concept is to engage the learner based on the concept of active learning ( Snyder, 2003).

## 3. The online Inverted Classroom Model (oICM)

**Figure 1.**
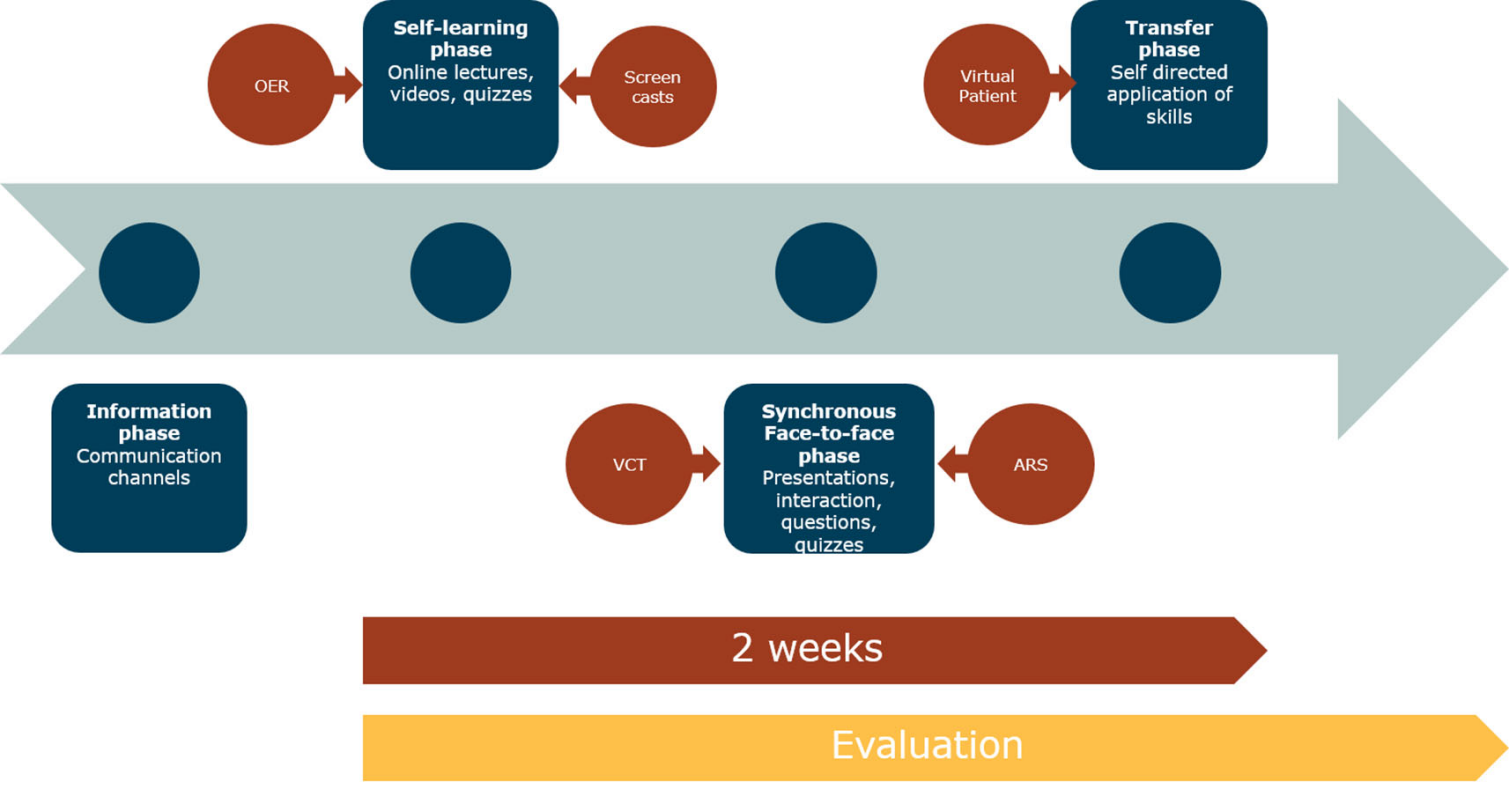
The online inverted classroom model (oICM)

Faced with the challenges of the corona pandemic, universities had to adapt their curricula to online methods quickly. The traditional ICM proposed by Tolks *et al.* was adapted purposefully for the new online approach (see Figure 1) ( Tolks *et al.*, 2016).

This blueprint was mainly designed for educators without prior experience with the ICM, who already use blended learning or especially the traditional ICM. However, teachers with no prior experiences can also use this blueprint but probably will require more preparation and maybe guidance.

Online teaching can be differentiated in synchronous vs. asynchronous delivery modes. In synchronous teaching learners and teachers meet at the same time with video conferencing tools. In asynchronous teaching, learning media is produced and, for example, provided via a learning management system (LMS), where learners consume content *ad libitum.* The advantages and disadvantages of both modes are listed in Table 1.

**Table 1.** Advantages and disadvantages of synchronous and asynchronous delivery modes

	Advantages	Disadvantages
Synchronous	Instant interaction for communication and collaboration Less isolation Corporate feeling Fast reactions to uncover misunderstandings	Rigid time window Fast internet connection needed Sophisticated hardware and software needed Quiet and acoustically as well as brightness adequate environment needed
Asynchronous	content can be downloaded everywhere any time - i.e. flexibility for time and place Students could spend more time on task - e.g. review material Permanent availability	Very limited communication and collaboration Missing social interaction Misunderstandings undetectedHuge amount of data Copyright and privacy violations if content is stored publicly available

As mentioned before, the most important part of the oICM is to maintain learner activation. Active rather than passive learning requires the involvement of students in the learning process, resulting in a more intense learning experience that goes beyond memorization ( Snyder, 2003). Active learning leads to more sustainable knowledge acquisition and promotes problem-solving skills ( Chi and Wylie, 2014). Furthermore, activities and interaction create a more learner-centered environment and are based on constructivist teaching rather than a direct instructional, mainly unilateral approach ( Salmon, 2013). Designing clear learning outcomes for online learning is just as important as for face-to-face environments.

### 3.1. Pre-phase

Before starting to teach, the learners have to be informed about the concept and process of the oICM, technical requirements, learning outcomes and expectations. It is important to offer the possibility to co-shape (e.g. a short pre-survey) and to use communication channels for your learners. This can be done via your LMS or by e-mail or a short synchronous session. At this stage students should be able to explain why they want to participate. What makes the course interesting? Therefore, special attention should be paid to the learning experience from the beginning ( *e.g.* intuitive learning tools, clear structure of the learning process).

### 3.2. Online Self-learning phase

As for ICM, in this asynchronous phase, it is important to provide learning material that targets the needs of your students. Students are used to learn from videos so we recommend using videos as a source for online learning ( Hurtubise *et al.*, 2013) as well as interactive tools. There are a fast variety of different methods and tools available. For some recommendations, you can look at the list of the digital tools (German) from the committee “Digitalization” of the German Association for Medical Education (GMA): tinyurl.com/ADTLLGMA

There are many ways to create learning content using videos. We would endorse three different approaches.

The easiest way to create great videos is by using screencasts. Screencast software ( *e.g.* Screen-O-Matic, SnagIt) allows you to record your presentation together with your speech. You may also include a video of you while talking. These programs are mostly free of charge and easy to use. Be aware that the presentation does not have to be perfect from a technological and rhetorical point of view. A study by Carpenter *et al.* (2013) has shown, that, with imperfect presentations, learners have a higher cognitive activity and a better knowledge retention rate compared to perfect presentations. Care should be taken to ensure that presentations are not overloaded with content and complex animations, but are structured, in high-resolution and reduced to essential elements. This applies especially to online teaching. To support the learning process, a handout could be useful. You can also use existing video tools that help you create innovative learning videos with cartoons (PowToon) or draw images with a digital pencil.

Another possibility is to record the speaker with a camera. This is often used in massive open online courses (MOOCs). These learning videos were analyzed in a study by Guo, Kim and Rubin (2014) regarding the engagement rate of students. To improve learner engagement, the following recommendations can help to improve the quality of the videos:

• Shorter videos are much more engaging
• Videos that intersperse an instructor’s talking head with slides are more engaging than slides alone
• Video produced with a more personal feel could be more engaging than high-fidelity studio screencasts
• Khan-style tablet drawings tutorials are more engaging than Powerpoint slides
• Videos, in which instructors speak fast and with enthusiasm, are more engaging
• Students engage differently with lecture and tutorial videos

A third approach is based on Open Educational Resources (OER). You can always use freely available learning content that is offered under an open-access license. Many universities offer their high-quality learning material including universities like Oxford, Harvard, Cambridge, or the Massachusetts Institute of Technology. Or you look at special databases *e.g.* Open Education EU to find suitable learning material.

During the online self-learning phase, quizzes engage the learners during and after the learning sessions and offer a way to assess their learning process. At the end of the self-learning phase, a final quiz should be implemented to assess the learning status of the group. If any problems or low rates in quiz occur, the teacher can address them in the upcoming online face-to-face phase. Most LMS provide tools for assessment and self-assessment.

### 3.3. Synchronous, online face-to-face phase

In an ICM setting the self-learning phase is followed by a face-to-face phase. One way to reduce the limitations of an online scenario such as low retention and engagement rates of students is to use synchronous online meetings. In oICM, this phase will take place online using video conferencing tools (VCTs), such as AdobeConnect, Zoom, or GoToMeeting. As in ICM, the session is moderated by the educator in real-time and in an interactive way. Depending on the number of students, this can be realized in form of interactive webinars or small group discussions making use of the small group rooms most VCTs offer.

In addition to VCTs and in order to engage the learner you can use various, easy-to-use, digital tools. Helpful tools are audience response systems (ARS) such as Kahoot!, Mentimeter, and/or Pingo or VCT integrated tools. Applying those programs, the facilitator can quiz students, create word clouds, rate questions, and use gamification approaches such as points, leaderboards, and badges ( Sailer and Homner, 2019). The (o)ICM offers a good structure to implement gamification aspects within the learning scenario ( Sailer and Sailer, 2020). Addressing questions with the help of ARS continuously activates participants to collaborate on the content and thus allow a more thorough reflection. While in traditional teaching sessions potentially more than half of all present participants are mentally distracted, ARS effectively counteracts this. Overall, they have a proven positive effect on learning outcomes ( Nelson *et al.*, 2012; Szpunar, Moulton and Schacter, 2013). In addition, the facilitator receives feedback from a large proportion of those present. A further benefit for the use of ARS in the oICM: Once an interactive quiz is used, learners are more likely to use the chat or audio connection for discussion.

This way, the moderator gets feedback, and it lowers the barrier for students to participate actively by talking or using the chat function. Case-based learning and problem-oriented learning can be easily facilitated by combining an online-meeting with collaborative online documents. For creating complex classifications, students might be asked to build a concept map ( *e.g.* Miro, MindMap or integrated whiteboard in ZOOM).

For students not attending the online synchronous meeting, the session can be recorded and uploaded to the LMS. In this case, every participant must agree to the recording and its use. However, we recommend including some additional homework to avoid a drop-out of the regular online sessions.

### 3.4. Transfer phase

The transfer phase deepens the learning outcome with another asynchronous learning session, where the learner will be challenged to apply their knowledge and transfer it to other content domains or contexts. This can be achieved with small projects students have to work on collaboratively, *e.g.* solving problems, virtual patients, virtual labs or let students develop cases or videos themselves.

## 4. Evaluation

After the oICM, it is also important to evaluate the process and outcome of the curriculum. Especially the online face-to-face phase should be critically assessed in the evaluation as it is a new approach within the ICM concept. We recommend integrating the evaluation into the online face-to-face meeting to enhance the response rate.

There already exist validated tested evaluation questionnaires for traditional teaching sessions like lectures and seminars ( Perry and Smart, 2007; Schiekirka *et al.*, 2015). However, it is possible to develop a short evaluation questionnaire for oICM based on existing instruments of traditional teaching and online teaching. In Appendix 1, we provide some examples for items structured by different aspects of teaching.

## 5. Conclusion

The oICM is based on the traditional ICM. The focus is on synchronous digital teaching, the activation of learners, and making use of VCTs and ARS. The oICM concept supports educators in transferring their previous face-to-face teaching into online teaching in a structured and meaningful way.

An advantage of the oICM concept is that after returning to face-to-face teaching nearly all the phases can be used without changes. The synchronous phase can be easily transformed into face-to-face teaching such as small group sessions or seminars including transferring the concept and content for using ARS.

Thus, with a few modifications of the existing ICM concept and with this easy-to-use approach presented here, it is possible to significantly improve digital teaching and support students in their learning process. Another positive aspect is that the oICM can be used even after the pandemic as ICM. The online learning material provided can be used during the self-learning phase for the teaching concept. The online ARS can be used during the face-to-face phase to activate the learners. Before, during and after the CoVid19 pandemic, oICM is an innovative approach to shape digital teaching and learning processes.

We hope that with this blueprint educators will be able to develop their own oICM teaching concepts, that also can be used after the pandemic and additionally build concepts that are feasible for the future. We would like to encourage all educators to invest more time in their teaching concept now and use this blueprint so that these challenging times have a positive impact on teaching.

## Take Home Messages

• The online Inverted Classroom Model (oICM) is an innovative and easy to use approach to shape digital teaching and learning processes during and after the CoVid19 pandemic. With a few modifications of an existing ICM concept educators can transform their face-to-face teaching activities into digital teaching.
• The proposed online Inverted Classroom Model (oICM) includes the following phases: (1) pre-phase, (2) online self-learning-phase, (3) synchronous online face-to-face phase, (4) transfer-phase, (5) evaluation.
• The most important part of this concept is to maintain learner activation with synchronous digital teaching using video conferencing tools (VCRs) and audience response systems (ARS).

## Notes On Contributors

**Dr. Daniel Tolks** studied public health and is a post-doc researcher at the chair for medical education at the Medical Faculty of LMU Munich and at the Centre for Applied Health Promotion at the Leuphana University Lüneburg. His research interests are technology enhanced learning, gamification and serious games for health. He is chair of the commitee “Digitalization” of the German Association for Medical Education and the German Network Gamification and Serious Games for Health. ORCID iD: https://orcid.org/0000-0001-8597-5189

**Bernd FM Romeike**, MD, MME, is medical educator at the University Medical School Rostock, Germany and clinical neuropathologist. He received his MD in Frankfurt M. in 1994, a habilitation for neuropathology in 2009 at the Homburg Medical School, and a Master of Medical Edication in Heidelberg in 2017. ORCID iD: https://orcid.org/0000-0002-9693-3870

**Jan P. Ehlers**, DVM, MA, FTA, is a veterinarian, instructional designer and medical educator. Her holds the chair for didactics and educational research in healthcare at the medical department and serves as vice president of Witten/Herdecke University, Germany. His research interests are digital transformation of health care, technology enhanced learning and higher education didactics. ORCID iD: https://orcid.org/0000-0001-6306-4173

**Sebastian Kuhn**, MD, MME is a Orthopedics and Trauma surgery and Medical Educator. His research interest on digital transformation an artificial intelligence in healthcare and education. ORCID iD: https://orcid.org/0000-0002-8031-2973

**Dr. Christin Kleinsorgen**, is a veterinarian and research associate in the Centre for E-Learning, Didactics and Educational Research at the University of Veterinary Medicine in Hannover, Germany. ORCID iD: https://orcid.org/0000-0003-1086-1691

**Johanna Huber**, MPH is a research associate and post-doc researcher at the chair for medical education at the Medical Faculty of LMU Munich and works in the field of evaluation studies, questionnaire construction and validation, graduate studies with a focus on the scientific, professional and social skills development, and health research capacity development.

**Martin Fischer**, MD, MME, FAMEE, is an internist, endocrinologist, and medical educator. He holds the chair for medical education and serves as the Assoc. Dean of Clinical Studies at the Medical Faculty of LMU Munich, Germany. His research interests are clinical reasoning skills, faculty and curriculum development, and technology-enhanced learning. ORCID iD: https://orcid.org/0000-0002-5299-5025

**Christoph Bohne** is a research scientist and specialist for educational technology at the Brandenburg Medical School Theodor Fontane.

**Luisa Merz** has a Master’s degree in Sociology and works at the Center for International Health at LMU University Hospital as a coordinator of training programs and courses in the field of Global Health with a focus on e-learning and international courses. In addition to various e-learning projects, she has coordinated a blended learning master program in the field of International Occupational Safety and Health.

**Michael Sailer** is a postdoctoral scholar at the Chair of Education and Educational Psychology at LMU Munich. He is currently conducting research about gamified learning, simulation-based learning and the use of technology in classrooms. ORCID iD: https://orcid.org/0000-0001-6831-5429

**Inga Hege**, MD, MCompSc, is an Associate Professor for Medical Education at the Medical School, University of Augsburg, Germany. ORCID iD: https://orcid.org/0000-0003-4335-5162

## Figures and Tables

**Appendix 1:** Evaluation questionnaire

Aspect of teaching	Items	Scale/ Answer format
Organization and technology	It was clearly communicated from the beginning how the online course format will look like.	5 Point Likert Scale for agreement n.a.
	Technical problems occurred before or during the teaching session.	5 Point Likert Scale for agreement n.a.
	If yes, please briefly describe the technical problems:	Free text question
Learning content	Which online activities (forms of learning/learning materials) were used in the teaching session?	Online communication (e.g. Forum) Live lesson (e.g. via Zoom or Adobe Connect) Selflearning test (e.g. MC questions, AMBOSS question sessions) Instructional video Podcast (audio record) Script (e.g. PPTslide sets, summaries) Online (group) task
	I have participated in live lessons or online communication ( *e.g.* forum).	Completely Partially Not at all n.a.
	I have used/worked with the provided online learning materials.	Completely Partially Not at all n.a.
	The learning content of the provided materials was understandable.	5 Point Likert Scale for agreement n.a.
	Which online activities were particularly helpful for you and why?	Free text question
Didactics and support	The learning goals that I was supposed to achieve were clarified at the beginning.	5 Point Likert Scale for agreement n.a.
	The online activities in this teaching session had clear tasks and goals.	5 Point Likert Scale for agreement n.a.
	During online activities the teacher supported me well.	5 Point Likert Scale for agreement n.a.
	The lecturer responded well to questions and suggestions.	5 Point Likert Scale for agreement n.a.
	I was encouraged to critically reflect upon the contents taught.	5 Point Likert Scale for agreement n.a.
Learning success	I can give an overview of the contents of the teaching session.	5 Point Likert Scale for agreement n.a.
	Measured by my previous knowledge, I learned a lot during the teaching session.	5 Point Likert Scale for agreement n.a.
Overall rating	I particularly liked the following aspects of the teaching session:	Free text question
	The following three aspects should be improved:	Free text question
	Overall, I rate the attended teaching session with:	5 Point Scale: excellent to insufficient n.a.
