# Supplementary material for: The online inverted classroom model (oICM). A blueprint to adapt the inverted classroom to an online learning setting in medical and health education
Source: MedEdPublish (2016). 2021 Sep 29;9:113. Originally published 2020 May 28. [Version 2] doi: 10.15694/mep.2020.000113.2 (PMC10702666; doi:10.15694/mep.2020.000113.2)
Supplement: Peer review history

# Reviewer report: Reviewer response for version 2

**DOI:** 10.21956/mep.20270.r31463

**Published:** 2021-10-05

**Authors:** Stijntje Dijk

**Affiliations:** 1 Erasmus Medical Center

This review has been migrated. The reviewer awarded 4 stars out of 5 The authors provided a helpful blueprint to medical teachers seeking to use a flipped-classroom approach in an online environment setting. In this helpful paper, they lay out steps to consider, from rationale, preparation of asynchronous learning materials, the synchronous session, a short note on transfer of knowledge, and the evaluation. The paper provides those with an interest in the topic with a collection of tips and tricks mostly regarding the preparation of learning materials. Additionally, the authors are to be congratulated on the diverse group of contributors to this paper.I have some notes in reaction to the paper, none of which require a full revision of the paper by themselves, but could be taken into account for future work / planned revisions:- The paper does not contain a section / explanation / reflection on how the blueprint came to be. Although it is a review of literature much more than empirical research, the paper may benefit from a section added to the introduction or elsewhere describing the methodology of writing the paper would be helpful.- The paper focusses mostly on the presentation of information (short/long videos, powerpoint vs alternatives, the use of quizzes). When thinking about the creation of learning content, a crucial part of the design of educational material is also the actual content: What type of learning objectives/topics could be more / less suited for this format? Crucially, next to what formats/platforms/tools to use, how can the facilitator assess that students prepared for the session and correctly understood the material? What happens in the transfer phase, how can a facilitator guide students to ensure that knowledge is further deepened and not lost? I would consider two approaches to these rising questions: to either specify that the blueprint specifically aims to guide choices in preparation of learning material rather than all aspects of flipped learning, or to dedicate additional attention to the assessment of learning.- A short note on terminology: I had difficulty finding out what "Khan-style tablet drawings" were, I assume this is a reference to the platform Khan Academy? Additionally, Powtoon is spelled as Powton.I would like to thank the authors for providing this blueprint, which can be very helpful to those seeking tools and tips to make their online teaching more engaging.

Reviewer Expertise:

NA

No decision status is available

# Reviewer report: Reviewer response for version 2

**DOI:** 10.21956/mep.20270.r31464

**Published:** 2021-10-05

**Authors:** Ken Masters

**Affiliations:** 1 Sultan Qaboos University

This review has been migrated. The reviewer awarded 4 stars out of 5 The authors have addressed my main concerns about Version 1 of the paper, and have clarified several concepts, and have also ensured that novices would not have unrealistic expectations of the task.Although the implementation would require adaptation to specific circumstances, the paper serves as a useful guide for moving the inverted classroom to the online environment.

Reviewer Expertise:

NA

No decision status is available

# Reviewer report: Reviewer response for version 1

**DOI:** 10.21956/mep.19005.r27264

**Published:** 2020-10-16

**Authors:** Ken Masters

**Affiliations:** 1 Sultan Qaboos University

This review has been migrated. The reviewer awarded 3 stars out of 5 An interesting paper on how to adapt the inverted classroom to an online learning setting in medical and health education. This is a useful contribution for teachers struggling to cope with the new demands of online teaching. The Appendix is also a useful sheet, especially for novices or for those who do not wish to work through the (sometimes) very demanding industry-standards. There, however, a few issues that need to be addressed.• The paper says that “This blueprint is designed for teachers without any prior experience with the ICM, but also for those who already teach in a traditional ICM”. I think, however, if someone has never used ICM before, then there would have to be far more training than this blueprint, and I think the authors are a little blasé about just how difficult it is for people to transition from old-style didactic teaching to any ICM process (if it were easy, everybody would be doing it). So, I think that the authors should make it clear that this would serve those who already use ICM in face-to-face teaching; those who don’t would do well to team up with a person experienced with ICM, or, if they have access to decent educational support structures, then consult and be guided by those. • The “Pre-phase” and “Transfer phase” could do with some expansion and examples, especially as this paper would be mostly beneficial to novices.Smaller issues• I am presuming that the heading “3.1 Pre-phase” corresponds to the portion “Online information Phase” in Figure 1. If so, this should be made clearer (either use the same words, or refer directly to it); if not, then it should be explained.• Although the abbreviations in Figure 1 are explained later in the text, they should also be given as a key to Figure 1. This is in keeping with the standard process of expanding an abbreviation the first time it is used anywhere in the paper. I look forward to Version 2 of this paper in which these issues are addressed.

Reviewer Expertise:

NA

No decision status is available

# Reviewer report: Reviewer response for version 1

**DOI:** 10.21956/mep.19005.r27263

**Published:** 2020-06-11

**Authors:** Nagwa Hegazy

**Affiliations:** 1 Menoufia university

This review has been migrated. The reviewer awarded 4 stars out of 5 Informative great work. great descriptive guide with a good evaluation checklist.thanks

Reviewer Expertise:

NA

No decision status is available

# Reviewer report: Reviewer response for version 1

**DOI:** 10.21956/mep.19005.r27265

**Published:** 2020-06-11

**Authors:** Hebat Allah A. Amin

**Affiliations:** 1 Department of Pathology, Helwan University, Faculty of Medicine

This review has been migrated. The reviewer awarded 4 stars out of 5 Thank you for your informative, well-structured guide. I've benefited from reading this interesting experience. The evaluation table added value to the work. Much appreciated.

Reviewer Expertise:

NA

No decision status is available

# Reviewer report: Reviewer response for version 1

**DOI:** 10.21956/mep.19005.r27266

**Published:** 2020-05-28

**Authors:** P Ravi Shankar

**Affiliations:** 1 American International Medical University

This review has been migrated. The reviewer awarded 4 stars out of 5 This is an interesting manuscript describing how an inverted classroom model can be adapted for an online approach during the pandemic. The paper has been written very well and is easy to follow The questionnaire used to evaluate the model shown in the Appendix is also easy to use. I am not sure what an AMBOSS question mentioned in Appendix 1 is. I enjoyed reading this well-written manuscript. The article will be of interest to all educators.

Reviewer Expertise:

NA

No decision status is available
